# Three latent profiles of learning engagement: a multicenter study based on vocational nursing students in China

**DOI:** 10.3389/fpsyg.2026.1765169

**Published:** 2026-03-31

**Authors:** Qian Jiang, Yiqian Fang, Chaoya Hu, Juan Du, Weiwei Zhang, Qin Xu, Yufan Zhang, Xiaoxia Lin, Qian Yang

**Affiliations:** 1 School of Nursing, Chengdu Medical College, Chengdu, Sichuan, China; 2 ICU, Chengdu Second People’s Hospital, Chengdu, Sichuan, China; 3 Department of Nursing, The Second Affiliated Hospital of Chengdu Medical College, Nuclear Industry 416 Hospital, Chengdu, Sichuan, China; 4 School of Public Health, Chengdu Medical College, Chengdu, Sichuan, China; 5 Southwest Medical University Affiliated Hospital Health School, Luzhou, Sichuan, China; 6 Hemodialysis Center, The First Affiliated Hospital of Chengdu Medical College, Chengdu, Sichuan, China

**Keywords:** academic emotions, academic self-efficacy, latent profile analysis, learning engagement, nursing students

## Abstract

**Introduction:**

Learning engagement refers to the sustained and positive psychological state that students exhibit during the learning process. It significantly influences nursing students’ academic performance, knowledge acquisition, higher-order thinking skills development, and the assessment of their career potential. However, due to the influence of traditional teaching models, the current level of learning engagement among nursing students in Chinese vocational colleges is generally low, and the differences between various student groups have not yet been fully explored.

**Methods:**

Using convenience sampling, a multicenter cross-sectional survey was conducted from January to March 2025 among nursing students from five vocational colleges in Sichuan Province. The study employed the Learning Engagement Scale, Academic Self-Efficacy Scale, and Academic Emotions Scale for College Students.

**Results:**

A total of 2,624 nursing students were included. Learning engagement profiles were categorized into: active-deep type (19.4%), moderate-participatory type (64.1%), and passive-compliant type (16.5%). Gender, place of origin, student leadership experience, academic performance, academic emotions, and self-efficacy for learning behaviors were significant influencing factors (*p* < 0.05).

**Discussion:**

The learning engagement of vocational nursing students is at a moderate level and exhibits group heterogeneity, which can be categorized into three potential profile types. Factors such as gender, place of origin, student leadership experience, academic performance, academic emotions, and self-efficacy in learning behaviors are influential in determining different categories of learning engagement.

## 1 Introduction

With the promulgation of the *Implementation Opinions on the Construction of First-Class Undergraduate Courses in 2019*, China’s higher education explicitly incorporated “learning engagement” into the national policy framework, reflecting a student-centered development approach aimed at unlocking students’ intrinsic potential and motivation while emphasizing their all-around development in moral, intellectual, physical, aesthetic, and labor education. This shift has made college student engagement not only a new trend in higher education quality assessment but also an increasingly hot topic of common concern in the global higher education community. The core of higher education quality lies in the quality of student development—that is, the comprehensive enhancement of students’ knowledge, abilities, and competencies throughout the learning process. Therefore, higher education quality evaluation should not only focus on students’ final academic performance but also pay attention to their psychological state, level of initiative, and degree of engagement during the entire learning process.

As a burgeoning research focus at the intersection of positive psychology and education, learning engagement is a multi-dimensional and multi-level concept. Its earliest origins can be traced back to 1984, when Astin (1984) proposed that learning engagement refers to the physical and psychological energy students invest in learning activities and its close relationship with their personal development. This concept primarily encompasses students’ demographic characteristics and family backgrounds, thereby laying the behavioral and energetic foundation for learning engagement. Building on this groundwork, Newmann (1992) further proposed “learning engagement” as a key indicator for evaluating students’ learning processes and educational quality, promoting the application of this concept in educational assessment. From the perspective of higher education practice, Kuh (2001) conceptualized learning engagement as the time and energy students devote to meaningful academic activities, as well as the sustained effort they exert to achieve educational goals. This perspective emphasizes that learning engagement is the product of the interaction between individual student factors and institutional environmental resources, including faculty support. With the deepening influence of positive psychology, Schaufeli et al. (2002) conceptualized learning engagement from a psychological perspective as a positive and persistent psychological state exhibited by students in academic activities, characterized by three core dimensions: vigor, dedication, and absorption. This definition marked a shift in the focus of learning engagement research from external behavioral observation to internal psychological experience, emphasizing it as an integrated psychological construct that combines emotional pleasure, cognitive immersion, and behavioral activation. Since then, scholars both domestically and internationally have conducted continuous and in-depth research on learning engagement, gradually establishing it as an important theoretical perspective for understanding student learning quality, predicting academic outcomes, and guiding educational practice.

Previous research has found that learning engagement is influenced by both internal and external factors, which can be categorized into school, individual, and family aspects (Guo, 2024). School-related factors include teacher-student relationships, classroom climate, school belonging, and teacher support (Shao et al., 2025). Individual-related factors encompass students’ degree of major identification, personality traits, academic emotions, learning motivation, learning interest, and self-efficacy (Wu et al., 2020; Mohamed Mohamed Bayoumy and Alsayed, 2021). Family-related factors involve parental companionship and parenting styles (Zhu et al., 2024).

As a critical academic psychological state, learning engagement not only profoundly affects the mastery of professional knowledge and clinical skills, but also exerts a far-reaching impact on an individual’s long-term career trajectory and the future quality of clinical nursing (Chen and Zhang, 2023). High learning engagement directly enhances learning outcomes by increasing course satisfaction and academic performance (Lam et al., 2012). It also significantly predicts future career development potential by fostering professional competence, occupational identity, and adaptability (Jiang, 2020). At the cognitive level, it promotes the formation of higher-order thinking skills such as critical and innovative thinking (Zhao, 2023), and by strengthening metacognitive ability, it heightens students’ awareness and control over learning and problem-solving. This process gradually internalizes as self-regulated learning capacity, ultimately enabling a fundamental shift from “episodic learning” to “lifelong learning” (Wijnen-Meijer, 2020; Wang et al., 2021).

Nursing, as a crucial component of the medical education system, directly influences the development level of healthcare services through the quality of its talent cultivation. Nursing is an important component of the medical education system, and the quality of its talent development is directly related to the overall level of the healthcare service system. Vocational nursing students, as a vital reserve force for the future healthcare workforce, warrant particular attention regarding their learning engagement. Existing research indicates that the overall learning engagement of vocational nursing students is at a moderate level (Liao et al., 2024). Compared to undergraduate nursing students, vocational nursing students face a shorter academic duration, a curriculum structure more focused on practical skills, generally lower academic self-efficacy, and the dual pressures of early clinical internships and career orientation, making them more susceptible to academic burnout, learning anxiety, and procrastination (Cao et al., 2025; Yang et al., 2025), which severely constrain their learning engagement.

However, current research in nursing education, both domestically and internationally, predominantly focuses on undergraduate and higher-level nursing students, with clearly insufficient attention paid to the important student group of vocational nursing students. Among the limited related studies, most remain at the level of describing overall engagement characteristics and lack in-depth analysis of the heterogeneity within the vocational nursing students population. This research gap not only limits a deeper understanding of the diversity of learning behaviors and their influencing mechanisms among vocational nursing students but also results in a lack of precise evidence for educational interventions targeting this group.

In light of this, this study aims to employ latent profile analysis to systematically identify the differentiated category structure of learning engagement among vocational nursing students and further explore significant differences among different categories of students in factors such as academic self-efficacy and academic emotions. The findings are expected to provide empirical evidence for constructing a stratified and categorized learning support system, ultimately facilitating a paradigm shift from “general education” to “precision intervention” to effectively enhance the quality of nursing diploma talent cultivation.

## 2 Methods

### 2.1 Participants

From January to March 2025, this study employed a convenience sampling method to select nursing students from five vocational colleges in Sichuan Province as research participants. Inclusion criteria: (1) Vocational college students majoring in nursing; (2) Informed consent and voluntary participation; (3) Good communication skills and the ability to independently complete the questionnaire. Exclusion criteria: (1) Students who took a leave of absence, withdrew from school, or were unable to participate in the study for personal reasons during the research period; (2) Those diagnosed with severe physical illnesses (e.g., malignant tumors, severe cardiovascular or cerebrovascular diseases) or serious mental disorders (e.g., schizophrenia, major depressive disorder).

### 2.2 Research instruments

#### 2.2.1 General information questionnaire

Developed based on an extensive review of relevant literature and in-depth group discussions, the specific criteria include 12 items: age, gender, grade level, place of origin, whether nursing was the first-choice major, whether serving as a student leader, whether receiving scholarships, Academic performance, whether an only child, family economic status, father’s education level and mother’s education level.

#### 2.2.2 Learning engagement scale

The scale was developed by Schaufeli et al. (2002) and later revised by Chinese scholar Fang et al. (2008). It consists of three dimensions (vigor, dedication and absorption) with a total of 17 items, rated on a 7-point Likert scale. Higher scores indicate a higher level of learning engagement in individuals. The scale demonstrates good reliability and validity, with Cronbach’s *α* coefficients ranging from 0.82 to 0.95.

#### 2.2.3 Academic self-efficacy scale

The scale was developed by Liang (2000), based on dimensions from the Academic Self-Efficacy Questionnaire originally designed by Pintrich and De Groot (1990). It consists of two dimensions: self-efficacy in learning ability and self-efficacy in learning behavior, comprising a total of 22 items. The scale uses a 5-point Likert rating system, with higher scores indicating greater self-efficacy. The Cronbach’s *α* coefficients for this scale range from 0.752 to 0.82.

#### 2.2.4 College students’ academic emotions scale

This scale was developed by Ma (2008), assessing academic emotions across four dimensions: negative high-arousal (shame, anxiety, and anger), positive high-arousal (interest, happiness, and hope), negative low-arousal (disappointment and boredom), positive low-arousal (pride and relaxation). The scale comprises 88 items in total. Its Cronbach’s α coefficients range from 0.641 to 0.887, and the test–retest reliability ranges from 0.563 to 0.866.

### 2.3 Ethical considerations

This study has been approved by the Ethics Committee (Protocol No: 2024NO.16, approved: 4/22/2024) and strictly adheres to the ethical principles of the Declaration of Helsinki. All participants provided written informed consent.

### 2.4 Data collection

This study utilized Questionnaire Star (an online survey platform) to distribute electronic questionnaires for data collection. First, the researchers thoroughly explained the study’s purpose, methods, confidentiality measures, voluntary participation, and other ethical considerations to the school administrators. After obtaining approval from the school’s ethics review board and the administrators’ consent, an electronic questionnaire link and an electronic informed consent form were distributed to eligible nursing students via class groups. All questions were set as mandatory to prevent omissions, and IP address restrictions were implemented to allow only one submission per device, ensuring data uniqueness. After questionnaire collection, a data verification team consisting of two uniformly trained researchers screened valid responses based on predefined criteria: excluding questionnaires with abnormal completion times (<3 min or >20 min), patterned responses (e.g., consecutive identical choices), or logical inconsistencies to ensure data authenticity and validity.

### 2.5 Statistical methods

Latent profile analysis (LPA) was conducted using Mplus 8.3 to identify distinct profiles of nursing students’ learning engagement. Models with 1 to 5 latent profiles were sequentially estimated, and the optimal model was selected based on the following three types of fit indices. ① Information criteria: The Akaike Information Criterion (AIC), Bayesian Information Criterion (BIC), and sample-size-adjusted BIC (aBIC) were used, with lower values indicating better model fit (Nylund et al., 2007). ② Classification accuracy: The entropy value was used to evaluate classification precision, where values closer to 1 indicate higher accuracy. An entropy value of 0.8 suggests classification accuracy exceeds 90%. ③ Likelihood ratio tests: The Lo–Mendell–Rubin adjusted likelihood ratio test (LMRT) and the bootstrapped likelihood ratio test (BLRT) were employed to compare the fit between models with *k*-1 and *k* classes. *p* < 0.05 indicated that the k-class model was superior to the *k*-1 -class model (Kim, 2014). Data analysis was performed using SPSS 26.0. Normally distributed continuous variables were presented as mean ± standard deviation, and group comparisons were conducted using one-way ANOVA. Non-normally distributed continuous variables were expressed as median and interquartile range, with group comparisons performed using the Kruskal-Wallis H test. Categorical or ordinal data were summarized as frequencies and percentages, and group differences were assessed using the chi-square test or Kruskal-Wallis H test, as appropriate. Multivariate analysis was conducted using multinomial logistic regression, with the identified latent classes as the dependent variable and statistically significant variables from univariate analysis as independent variables. A significance level of *p* < 0.05 was considered statistically significant.

## 3 Results

### 3.1 General characteristics of the study participants

A total of 2,744 questionnaires were collected in this study. After excluding 120 invalid responses, 2,624 valid questionnaires were retained, yielding an effective response rate of 95.6%. The study included 2,624 nursing students aged 16–24 years, with a mean age of 19.15 ± 1.2 years. Among the participants, 688 (26.2%) were male and 1,936 (73.8%) were female. In terms of geographical background, 2,145 students (81.7%) were from rural areas. Regarding academic year distribution, 1,445 participants (55%) were first-year students, while 1,076 (41%) were in their second year. Additionally, 1,192 students (45.4%) reported nursing as their first-choice major.

### 3.2 Learning engagement, academic emotions, and academic self-efficacy scores of vocational nursing students

The results demonstrated that vocational nursing students’ overall learning engagement score was 67.17 ± 15.16 points. Specifically, the vitality subscale scored 22.92 ± 5.86 points, dedication subscale scored 24.28 ± 6.09 points, and absorption subscale scored 19.97 ± 5.05 points. The scores for academic emotions and academic self-concept are presented in Table 1.

**Table 1:** Scores of learning engagement, academic emotions, and academic self-efficacy among vocational nursing students.

Variables		Minimum	Maximum	Score
Learning engagement	Vigor	6	42	22.92 ± 5.86
	Dedication	6	42	24.28 ± 6.09
	Absorption	5	35	19.97 ± 5.05
	Total score	17	119	67.17 ± 16.15
Academic emotions	Negative high arousal	27	135	85.69 ± 18.08
	Positive high arousal	19	95	64.51 ± 11.7
	Negative low arousal	23	115	67.3 ± 14.95
	Positive low arousal	19	95	59.89 ± 10.78
Academic self-efficacy	Self-efficacy for learning ability	11	44	24.71 ± 6.04
	Self-efficacy for learning behavior	11	44	24.85 ± 5.39
	Total score	22	88	49.56 ± 10.79

### 3.3 Results of latent profile analysis on learning engagement in vocational nursing students

Latent profile analysis was conducted using all items of learning engagement among vocational nursing students. The model fit indices are presented in Table 2. As the number of latent classes increased, the values of AIC, BIC, and aBIC gradually decreased while the Entropy value increased. However, when the number of classes reached four, the Entropy value began to decline. Comparative analysis revealed that the 3-class model demonstrated the lowest values for AIC, BIC, and aBIC, along with the highest Entropy value, indicating optimal balance between model fit and classification clarity. Based on these statistical indicators and the practical significance of classification, the 3-class model was identified as the optimal solution. Furthermore, the posterior probabilities for class membership were 0.977, 0.98, and 0.986 across the latent classes. This high probability range confirms the stability and reliability of the latent class classification, ensuring the accuracy and validity of subsequent analyses.

**Table 2:** Fit indices of latent profile analysis for learning engagement among vocational nursing students.

Model	AIC	BIC	aBIC	Entropy	LMR	BLRT	Probability (%)
1	140161.547	140361.223	140253.195				
2	125401.424	125706.812	125541.592	0.95	<0.001	<0.001	74.5/25.5
3	113615.389	114026.487	113804.077	0.964	<0.001	<0.001	19.4/64.1/16.5
4	108481.172	108997.981	108718.380	0.961	<0.001	<0.001	17.8/20.8/57.6/4.4
5	103147.241	103769.762	103432.969	0.958	<0.001	<0.001	5.1/20.4/51.7/18.4/4

### 3.4 Naming of latent profiles for learning engagement in vocational nursing students

**Figure 1:**
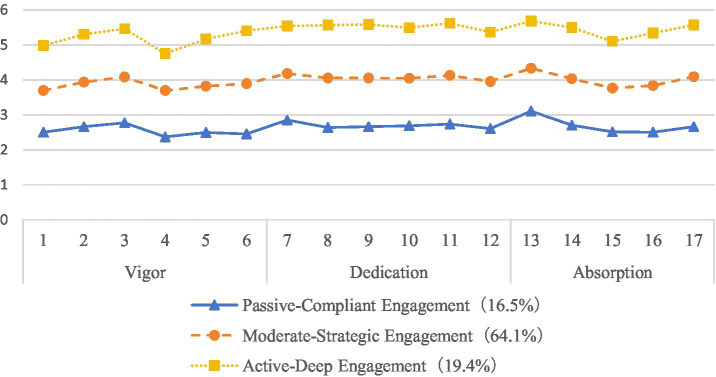
Latent profile analysis of learning engagement among vocational nursing students.

Based on the analysis of learning engagement scores across the three identified profiles, we constructed corresponding score distribution graphs (Figure 1), with item numbers on the *x*-axis and scores on the *y*-axis. The results revealed: The first profile (16.5%) demonstrated the lowest mean scores across all learning engagement items, with a total score of 44.89 ± 11.07. These students exhibited relatively passive learning behaviors and were therefore designated as the “Passive-Compliant” engagement type. The second profile (64.1%) showed intermediate mean scores, representing the majority population with a total score of 67.63 ± 5.56. Their learning engagement displayed moderate levels with fluctuating characteristics between active participation and occasional disengagement. This group was named the “Moderate-Participatory” engagement type. The third profile (19.4%) achieved the highest mean scores across all items, with a total score of 91.51 ± 11.77. These students demonstrated high-level engagement, characterized by proactive learning attitudes and deep learning involvement, thus being classified as the “Active-Deep” engagement type.

### 3.5 Univariate analysis results of different learning engagement profiles in vocational nursing students

The univariate analysis revealed that gender, geographical origin, first-choice major status, student leadership role, academic performance, family economic status, paternal education level, maternal education level, academic emotions, and academic self-efficacy were significant influencing factors for different learning engagement profiles (all *p* < 0.05). See Table 3 for details.

**Table 3:** Univariate analysis of latent classes in learning engagement among vocational nursing students.

Variables	Passive-compliant engagement *n* = 509	Moderate-strategic engagement *n* = 1,681	Active-deep engagement *n* = 434	*χ^2^/F*	*P*
Gender [*n*(%)]				222.08^a^	<0.001
Male	141(27.7)	397(23.6)	150(34.6)		
Female	368(72.3)	1,284(76.4)	284(65.4)		
Grade [*n*(%)]				8.356	0.079
Grade 1	271(53.2)	954(56.8)	220(50.7)		
Grade 2	223(43.8)	660(39.3)	183(44.5)		
Grade 3	15(2.9)	67(4)	21(4.8)		
Place of Origin [*n*(%)]				10.76^a^	0.005
Rural	441(86.6)	1,360(80.9)	344(79.3)		
Urban	68(13.4)	321(19.1)	90(29.7)		
First choice [*n*(%)]				9.51^a^	0.009
Yes	213(41.8)	755(44.9)	224(51.6)		
No	296(58.2)	926(55.1)	210(48.4)		
Student leadership roles [*n*(%)]				55.68^a^	<0.001
Yes	86(16.9)	497(29.6)	167(38.5)		
No	423(83.1)	1,184(70.4)	267(61.5)		
Scholarship [*n*(%)]				5.704^a^	0.058
Yes	98(19.3)	403(24)	108(24.9)		
No	411(80.7)	1,278(76)	326(75.1)		
Academic performance [*n*(%)]				83.99^a^	<0.001
Excellent	35(6.9)	191(11.4)	96(22.1)		
Good	305(59.9)	1,130(67.2)	265(61.1)		
Poor	169(33.2)	360(21.4)	73(16.8)		
Only child [*n*(%)]				0.952^a^	0.62
Yes	74(14.5)	272(16.2)	72(16.6)		
No	435(85.5)	1,409(83.8)	362(83.4)		
Family economic situation [*n*(%)]				13.35^a^	0.01
Poor	197(37.3)	584(34.7)	154(35.5)		
Average	312(61.3)	1,062(63.2)	260(59.9)		
Good	7(1.4)	35(2.1)	20(4.6)		
Father’s education level [*n*(%)]				20.35^a^	0.002
Primary school and below	218(42.8)	624(37.1)	149(34.3)		
Junior high school	205(40.3)	663(39.4)	161(37.1)		
Senior high school	59(11.6)	262(15.6)	86(19.8)		
Diploma and above	27(5.3)	132(7.9)	38(8.8)		
Mather’s education level [*n*(%)]				17.12^a^	0.009
Primary school and below	259(50.9)	786(46.8)	187(43.1)		
Junior high school	179(35.2)	579(34.4)	149(34.3)		
Senior high school	44(8.6)	216(12.8)	57(13.1)		
Diploma and above	27(5.3)	100(5.9)	41(9.4)		
Negative high arousal [^−^*x* ± *s*]	82.08 ± 20.76	86.77 ± 15.37	85.76 ± 23.19	13.259^b^	<0.001
Positive high arousal [^−^*x* ± *s*]	55.4 ± 11.9	64.86 ± 9	73.84 ± 12.63	377.5^b^	<0.001
Negative low arousal [^−^*x* ± *s*]	69.1 ± 15.88	67.6 ± 12.51	64 ± 12.88	14.72^b^	<0.001
Positive low arousal [^−^*x* ± *s*]	51.98 ± 11.12	59.97 ± 8.27	68.85 ± 11.8	367^b^	<0.001
Self-efficacy for learning ability [^−^*x* ± *s*]	25.4 ± 6.5	24.49 ± 4.1	24.75 ± 9.74	4.627^b^	0.1
Self-efficacy for learning behavior [^−^*x* ± *s*]	24.84 ± 5.73	24.58 ± 4.1	25.9 ± 8.4	10.53^b^	<0.001

^a^Represents the chi-square test; ^b^Represents the analysis of variance.

### 3.6 Multivariate analysis results of different learning engagement profiles in vocational nursing students

Using the three learning engagement types as outcome variables and incorporating statistically significant variables from the univariate analysis as independent variables, we conducted multinomial logistic regression analysis. The results demonstrated that gender, geographical origin, student leadership role, academic emotions, and academic behavioral self-efficacy were significant influencing factors for the latent profile classification of nursing students’ learning engagement (all *p* < 0.05). See Table 4 for details.

**Table 4:** Multivariate analysis of factors associated with learning engagement profiles among vocational nursing students.

Variables	Passive-compliant engagement vs. moderate-strategic engagement				Passive-compliant engagement vs. active-deep engagement			
	*B*	SE	*P*	OR(95%CI)	*B*	SE	*P*	OR(95%CI)
Negative high arousal	0.039	0.008	<0.001	1.04(1.02 ~ 1.06)	0.048	0.010	<0.001	1.049(1.03 ~ 1.07)
Positive high arousal	0.034	0.012	0.004	1.034(1.01 ~ 1.06)	0.065	0.016	<0.001	1.068(1.03 ~ 1.1)
Negative low arousal	−0.069	0.010	<0.001	0.933(0.92 ~ 0.95)	−0.094	0.012	<0.001	0.91(0.89 ~ 0.93)
Positive low arousal	0.058	0.012	<0.001	1.059(1.03 ~ 1.08)	0.135	0.016	<0.001	1.145(1.11 ~ 1.82)
Self-efficacy for learning ability	−0.019	0.017	0.265	0.981(0.95.1.02)	−0.003	0.023	0.909	0.997(0.95 ~ 1.04)
Self-efficacy for learning behavior	0.027	0.018	0.149	1.027(0.99 ~ 1.06)	0.079	0.024	<0.001	1.083(1.03 ~ 1.13)
Male	−0.040	0.131	0.763	0.961(0.74 ~ 1.24)	0.467	0.177	0.008	1.596(1.13 ~ 2.26)
Rural	−0.399	0.174	0.022	0.671(0.48 ~ 0.96)	−0.189	0.238	0.428	0.828(0.52 ~ 1.32)
First choice: yes	0.081	0.114	0.473	1.085(0.87 ~ 1.36)	0.169	0.157	0.283	1.184(0.87 ~ 1.61)
Student leadership roles**: yes**	0.495	0.142	<0.001	1.64(1.24 ~ 1.66)	0.828	0.181	<0.001	2.289(1.61 ~ 3.27)
Family economic situation								
Poor	−0.315	0.504	0.533	0.73(0.27 ~ 1.96)	−0.703	0.594	0.237	0.495(1.56 ~ 1.59)
Average	−0.406	0.496	0.413	0.667(0.25 ~ 1.76)	−0.892	0.578	0.123	0.41(0.13 ~ 1.27)
Father’s education level								
Primary school and below	−0.317	0.330	0.337	0.728(0.38 ~ 1.39)	0.186	0.443	0.675	1.204(0.51 ~ 2.87)
Junior high school	−0.200	0.318	0.528	0.818(0.44 ~ 0.53)	0.236	0.424	0.578	1.266(0.55 ~ 2.91)
Senior high school	0.007	0.333	0.982	1.007(0.52 ~ 1.94)	0.786	0.434	0.070	2.195(0.94 ~ 5.13)
Mather’s education level								
Primary school and below	0.291	0.342	0.394	1.338(0.69 ~ 2.61)	−0.315	0.443	0.478	0.73(0.31 ~ 1.74)
Junior high school	0.266	0.331	0.422	1.304(0.68 ~ 2.5)	−0.374	0.427	0.382	0.688(0.3 ~ 1.59)
Senior high school	0.441	0.352	0.210	1.555(0.78 ~ 3.1)	−0.339	0.449	0.451	0.713(0.3 ~ 1.72)
Academic Performance								
Excellent	0.485	0.233	0.038	1.623(1.03 ~ 2.57)	1.030	0.295	<0.001	2.801(1.57 ~ 5)
Good	0.187	0.127	0.140	1.206(0.94 ~ 1.55)	0.148	0.195	0.448	1.16(0.79 ~ 1.7)

## 4 Discussion

### 4.1 Status and heterogeneity in learning engagement levels among vocational nursing students

The results of the study showed that the learning engagement score of vocational nursing students was (67.17 ± 16.15) points, indicating an overall low-to-medium level. Which is lower than that of undergraduate nursing students reported by Liu et al. (2024) (79.83 ± 23.44) points, but higher than that of associate-to-bachelor nursing students reported by Gao et al. (2024) (52.49 ± 13.18) points. This discrepancy may reflect systematic differences in admission motivation, professional identity, and academic pressure among nursing students at different educational levels. Undergraduate nursing students are typically admitted through unified enrollment, possess greater autonomy in selecting their major, and exhibit relatively stable professional identity, thereby demonstrating higher levels of learning engagement. In contrast, some associate-to-bachelor nursing students may pursue further education primarily for practical purposes such as degree advancement, with utilitarian motivations playing a more significant role. This could, to some extent, limit their deep engagement at the emotional and cognitive levels. Meanwhile, vocational nursing students face direct competitive pressure in the job market. The dual burden of academic tasks and career preparation may subject them to a certain degree of stress, resulting in a learning engagement level that is “higher than that of associate-to-bachelor students but lower than that of undergraduate students.”

Latent Profile Analysis revealed three potential categories of learning engagement: the Active-Deep Type (19.4%), the Moderate-Strategic Type (64.1%), and the Passive-Compliant Type (16.5%). Regarding the items, item 4 (I can study for long periods without needing breaks) showed the lowest probability score. This suggests that nursing students may exhibit passive learning tendencies during their studies, resulting in relatively low willingness for sustained learning and difficulty maintaining prolonged, focused high-intensity study. Consequently, they require appropriate rest periods to maintain learning efficiency. Conversely, item 13 (When studying, I feel time passes quickly) demonstrated the highest probability score. This indicates that when engaged with interesting learning content, nursing students can generate strong intrinsic motivation that facilitates immersive learning states. Such deep engagement alters their time perception, leading to underestimation of actual elapsed time and creating the subjective experience of “time flying by.”

Among the different categories, the passive-coping type accounted for the lowest proportion (16.5%). Students in this group generally exhibited characteristics such as insufficient learning initiative, weak self-regulation abilities, and relatively negative academic emotions. Their learning engagement behaviors were often passive and task-avoidant. Therefore, it is recommended that educators enhance learning appeal through contextualized instructional design and guide students in adopting scientific time management strategies to gradually establish structured study habits. Additionally, efforts should be made to foster a supportive classroom atmosphere, leveraging teachers’ emotional support and peer modeling to stimulate their willingness to participate, thereby effectively improving learning engagement and academic performance (Lin et al., 2024).

The moderate-participatory type (64.1%) accounted for the largest proportion, reflecting that vocational nursing students, in an environment with shorter program durations and strong employment orientation, generally adopt a task-driven, efficiency-first adaptive learning strategy. Students of this type possess basic learning willingness and compliance, yet their level of engagement is easily influenced by external teaching arrangements, assessment methods, and immediate feedback, representing a typical transitional state between passive acceptance and deep engagement. Therefore, educators should emphasize process-oriented motivation by setting phased goals, providing timely feedback, and designing appropriately challenging tasks to guide students from “task completion” toward “meaningful engagement,” thereby maintaining and enhancing the quality and stability of their learning participation (Vermote et al., 2020).

Students of the active-deep type (19.4%) generally exhibit strong intrinsic learning motivation, effective self-regulation skills, and positive emotional experiences. They demonstrate sustained, focused, and strategic learning engagement. Therefore, educators should prioritize providing opportunities for extended learning and in-depth challenges, such as introducing inquiry-based projects, encouraging clinical reflection and research practice, and offering specific advanced feedback to support their progression toward higher levels of professional development.

### 4.2 Analysis of influencing factors on learning engagement in vocational nursing students

#### 4.2.1 Gender, geographical origin, student leadership role, and academic performance

The unordered multinomial logistic regression analysis indicated that gender, place of origin, whether serving as a student cadre, and academic performance influence the learning engagement of vocational nursing students. Specifically: the results demonstrated that male students showed a greater tendency to be classified into the Active-Deep profile, which aligns with the findings of Wang et al. (2016), but differs from Xu et al.’s (2014) conclusions. The reasons for this are: the differences in demographic characteristics, educational backgrounds of the study participants, as well as variations in research design and measurement tools may have led to different outcomes. This phenomenon suggests that the influence of gender on learning engagement is controversial and may be context-dependent. Future research needs to further control for potential confounding variables to more accurately reveal the relationship between gender and learning engagement.

Nursing students from rural areas are more likely to be categorized as passively coping types, which is consistent with the findings of Guo (2024). Urban nursing students generally have access to more abundant educational resources, diverse growth pathways, and higher-quality learning platforms, providing strong support for their learning engagement. Furthermore, urban families typically place greater emphasis on the holistic development of their children, offering both sufficient financial support and emotional care. This effectively sustains their learning enthusiasm and promotes proactive engagement. In contrast, nursing students from rural areas face multiple practical constraints, including relatively limited educational resources, insufficient family support, and lower societal expectations, all of which impact their level of learning engagement.

Students who serve as student cadres are more likely to belong to the active-deep or moderate-participatory types, which aligns with the findings of Wang (2021). Taking on multiple roles helps them develop stronger time management and planning skills, leading to better learning execution, lower procrastination tendencies, and significantly enhancing their level of learning engagement (Zhu, 2014). At the same time, it has been reported that through participating in and organizing various activities, this group generally exhibits a stronger sense of responsibility and self-motivation, while also accumulating more positive emotional experiences. These intrinsic motivations translate into concrete actions during the learning process, thereby promoting a higher level of learning engagement.

Students with excellent academic performance are more inclined to be categorized as actively deep and moderately involved types in terms of learning engagement. Nursing students with excellent academic performance typically hold positive ability beliefs, believing that their intelligence can be improved through effort, and thus worry less about potential failure. They are confident in their fundamental skills to complete their studies and meet the demands of the university environment, leading to fewer symptoms of academic burnout. This low-burnout state prompts them to proactively increase their study time and concentrate their energy on learning, thereby fostering a higher level of academic engagement (Nicita et al., 2025). Furthermore, strong academic performance is often accompanied by clear learning goals, sustained subject interest, and effective learning strategies. These factors collectively enhance students’ sense of goal achievement, making them more inclined to adopt a mastery goal orientation. This reduces their sense of alienation from academic pursuits, alleviates academic burnout, enhances learning satisfaction and personal accomplishment, and ultimately sustains and promotes academic engagement. Notably, this state of high academic engagement, reinforced by the positive feedback of improved academic performance, further consolidates students’ learning motivation, which in turn promotes the continuous improvement of their academic performance (Mohamed Mohamed Bayoumy and Alsayed, 2021).

#### 4.2.2 Academic self-efficacy

Academic self-efficacy refers to an individual’s judgment and confidence in their ability to successfully complete academic tasks (Bandura, 1977; Liang, 2000), encompassing two aspects: self-efficacy for learning ability and self-efficacy for learning behavior. Previous studies have found (Hayat et al., 2020) that academic self-efficacy is a core factor influencing nursing students’ learning motivation, cognitive abilities, and emotional states. It has a significant impact on shaping their learning beliefs and achieving academic goals, and is widely recognized as an important indicator for predicting students’ academic performance (Nazari et al., 2025).

The results of this study indicate that individuals with high learning behavior self-efficacy are more likely to be categorized as actively deep types, similar to the findings of Dogan (2015). Learning behavior self-efficacy refers to an individual’s self-assessment and subjective judgment of their ability to take effective measures and ultimately achieve learning goals through their own efforts (Liang, 2000). On one hand, nursing students with high learning behavior self-efficacy typically have a firm belief in their own learning abilities. They can not only accurately identify and fully utilize their personal advantageous resources but also possess learning strategies to meet academic requirements (Taghani and Razavi, 2022). This enables them to approach learning with interest and passion, view learning difficulties as opportunities for growth, and actively cope with them, thereby reducing academic burnout and demonstrating stronger willingness to challenge and persevere. This, in turn, continuously stimulates their intrinsic learning motivation and further enhances their level of learning engagement (Abdolrezapour et al., 2023; Firdausi et al., 2023). On the other hand, the enhancement of academic self-efficacy helps to strengthen nursing students’ sense of control and value recognition during the learning process, thereby promoting more positive emotional experiences. Academic behavioral self-efficacy can facilitate a more positive psychological state in nursing students by improving psychological resilience and regulating academic emotions. This positive emotional experience, in turn, translates into more sustained and focused learning engagement behaviors (Putwain et al., 2013).

Therefore, nursing educators should focus on providing positive guidance for vocational nursing students by adopting reinforcement systems and positive feedback (Alrabai and Alamer, 2024) to acknowledge students’ efforts and progress throughout the learning process. Such practices can stimulate their learning enthusiasm, confidence, and initiative, thereby fostering positive academic self-efficacy and further promoting learning engagement and academic performance. Additionally, educators can design brief mindfulness exercises tailored to academic contexts to help students better recognize and understand their rational selves. When students enter the learning space in a state of mindful awareness, they are often better able to regulate their sensory experiences, emotional responses, and expressive capabilities. This, in turn, facilitates the development of academic self-efficacy, which behaviorally manifests as enhanced concentration and an increased willingness to engage in learning (Leland, 2015).

#### 4.2.3 Academic emotions

Academic emotions refer to the emotional experiences associated with all learning-related activities (Pekrun et al., 2002). The results of this study show that academic emotions are influential factors in various categories of learning engagement. Among them, individuals with positive high arousal, negative high arousal, and positive low arousal are more likely to be categorized as actively deep and moderately involved types in terms of learning engagement, while those with negative low arousal are more likely to be categorized as passively coping types, similar to the findings of Fu et al. (2024). Based on Pekrun’s (2006) control-value theory, positive academic emotions not only enhance students’ learning motivation and goal-pursuit intentions but also encourage them to adopt more effective learning strategies and maintain sustained concentration, thereby forming a virtuous cycle of learning engagement (Tan et al., 2021). In contrast, negative academic emotions tend to trigger avoidance behaviors, manifesting as maladaptive strategies such as procrastination and distraction, ultimately reducing the level of learning engagement (Li et al., 2023). Furthermore, there exists a dynamic bidirectional relationship between academic emotions and learning engagement: positive emotions enhance learning engagement through a positive feedback mechanism, while high learning engagement further reinforces positive emotional experiences. Simultaneously, students can actively intervene in negative emotional states through emotional regulation strategies. This indicates that academic emotions not only directly shape learners’ cognition, motivation, and behavior but are also reciprocally influenced by academic performance and learning experiences, thereby forming an interactive reciprocal system of “emotion-behavior”(Huang and Cherng, 2021).

Therefore, nursing educators should pay close attention to the emotional changes of vocational nursing students, providing them with timely, specific, and positive feedback throughout the learning process. At the same time, educators should cultivate students’ dispositional mindfulness—characterized by a non-judgmental, open, and attentive awareness—to equip them with stronger emotional regulation skills (Sorrenti et al., 2025). This approach helps alleviate emotional distress during learning, relieve psychological discomfort such as learning burnout and anxiety, thereby laying a solid psychological foundation for sustained learning engagement. Additionally, innovative teaching methods such as blended learning, escape room pedagogy, or simulation-based education can be employed (González-de La Torre et al., 2024; Li et al., 2023; Yang et al., 2021). Specifically, the following instructional design could be implemented in professional courses: before class, short videos and self-assessment questions are distributed via an online platform to guide students in independent learning; during class, high-fidelity simulators are used to conduct group situational drills, incorporating an “escape room” mechanism that requires students to collaboratively complete clinical case reasoning and operational tasks within a set time limit; after class, online discussions and personalized feedback are provided to consolidate learning outcomes. These teaching designs, grounded in the principle of “learning through play” and supported by structured interaction and immediate feedback, help nursing students accumulate successful experiences in realistic contexts. This gradually fosters a positive learning cycle of “effort—feedback—reinforcement,” thereby cultivating sustained intrinsic motivation and achieving dual improvements in learning efficiency and engagement.

### 4.3 Limitations of this study

This study has the following limitations: First, in terms of research design, this study employed a cross-sectional survey, and its results can only reveal correlations between variables, not infer causal directions. Additionally, although latent profile analysis was used to identify different types of learning engagement, this method itself is limited in examining the mediating or moderating mechanisms of academic emotions or self-efficacy across different categories. Therefore, future research could adopt longitudinal tracking designs or cross-lagged models to further examine the causal pathways and dynamic influences of various factors on learning engagement. Furthermore, it is recommended that broader samples be used to systematically test the applicability and boundary conditions of the “affect-cognition-behavior” chain model across different student subgroups—such as those with varying educational levels or professional backgrounds—through structural equation modeling or multilevel linear analysis, thereby comparing the explanatory power of different theoretical pathways, such as chain, parallel, or interactive effects. Second, regarding sample representativeness, this study employed convenience sampling, with all participants coming from five vocational colleges in Sichuan Province, resulting in limitations such as a single geographical area and limited types of institutions. The sample showed a relatively high proportion of rural students and female participants, a phenomenon largely consistent with the actual demographic structure of nursing education in China. On one hand, the nursing profession has long been predominantly female, and this gender imbalance directly influences the composition of current student populations. On the other hand, factors such as social perceptions of the profession, employment orientation, and family economic considerations make nursing relatively more attractive to students from rural backgrounds, resulting in a lower proportion of urban students. Additionally, the study did not include nursing students who took a leave of absence or dropped out during the study period, which may have led to an incomplete analysis of the characteristics of “passively coping” students. Subsequent research could expand the sampling scope, use stratified random sampling methods, include nursing students from different geographical regions and educational levels (such as nursing programs in undergraduate universities) across the country, and cover special student groups to enhance the external validity of the sample. Third, this study only employed quantitative research methods and failed to deeply explore the psychological mechanisms and behavioral motivations behind the heterogeneity of learning engagement. Future research could combine qualitative research methods (such as phenomenological interviews and grounded theory) to conduct an in-depth exploration of the learning experiences, motivational drivers, and environmental influencing factors of different types of nursing students.

Despite the aforementioned limitations, this study still provides a significant reference for conducting precise interventions in the field of nursing education by revealing, for the first time through large-sample quantitative analysis, the group heterogeneity characteristics and key influencing factors of learning engagement among nursing students in vocational colleges. Subsequent research can build upon this study and further deepen the understanding of nursing students’ learning behaviors through mixed-methods research designs, thereby promoting the improvement of nursing education quality.

## 5 Conclusion

The findings of this study indicate that the overall learning engagement of vocational nursing students is at a moderate level, with significant group heterogeneity. They can be categorized into three types: active-deep engagement, moderate-participatory engagement, and passive-coping engagement. Gender, place of origin, whether serving as a student cadre, academic performance, academic emotions, and self-efficacy in learning behaviors are key factors influencing their classification into different learning engagement categories. In the future, nursing education administrators can develop targeted intervention strategies by improving nursing students’ academic emotional states and enhancing their self-efficacy in learning behaviors, so as to effectively promote their level of learning engagement.

## Data Availability

The raw data supporting the conclusions of this article will be made available by the authors, without undue reservation.
